# Nutrient loading as a key cause of short- and long-term anthropogenic ecological degradation of the Salton Sea

**DOI:** 10.1038/s41598-024-82633-y

**Published:** 2024-12-28

**Authors:** Caroline Hung, Charles Diamond, Ryan Sinclair, Meng-Chen Lee, Michael Stenstrom, Mara A. Freilich, Quinn Montgomery, Consuelo Marquez, Timothy W. Lyons

**Affiliations:** 1 Department of Earth and Planetary Sciences, University of California, Riverside, CA 92521 USA; 2 School of Public Health, Loma Linda University, Loma Linda, CA 92350 USA; 3 Department of Civil and Environmental Engineering, University of California, Los Angeles, CA 90095 USA; 4 Division of Applied Mathematics and Department of Earth, Environmental, and Planetary Sciences, Brown University, Providence, RI 02912 USA; 5 CA, USA

**Keywords:** Element cycles, Environmental impact

## Abstract

The Salton Sea (SS), California’s largest inland lake at 816 square kilometers, formed in 1905 from a levee breach in an area historically characterized by natural wet-dry cycles as Lake Cahuilla. Despite more than a century of untreated agricultural drainage inputs, there has not been a systematic assessment of nutrient loading, cycling, and associated ecological impacts at this iconic waterbody. The lake is now experiencing unprecedented degradation, particularly following the 2003 Quantification Settlement Agreement—the largest agricultural-to-urban water transfer in the United States. Combined with high evaporation rates, reduced inflows have led to rapid lake shrinkage, with current maximum depths of only 10 m. Here we report distinct temporal and spatial patterns for nutrient dynamics at the SS for two decades spanning the period before and after major water transfer agreement. While external nutrient loading remains relatively consistent year-round, internal cycling varies seasonally. Winter exhibits high total phosphates and nitrate levels due to reduced primary productivity, with lower ammonium concentrations from increased oxygenation. Summer conditions shift to decreased phosphate and nitrate levels from enhanced primary production, sustained partly by internal phosphorus release from sediments during anoxic periods. Although N:P molar ratios can exceed 50:1 to 100:1 (far above the Redfield ratio of 16:1), phosphorus consistently remains at hypereutrophic levels (> 0.05 mg/L) challenging previous assumptions of phosphorus limitation. Post-2020 data show disrupted stratification patterns. Despite higher oxygen levels in bottom waters compared to 2004–2009, overall water column oxygenation has declined, reflecting altered hydrodynamics in the shallowing lake. These changes have intensified environmental challenges stemming from cultural eutrophication including harmful algal blooms, threatening both ecosystem and public health. Effective remediation will require significant reduction in external nutrient loading through constructed wetlands and/or treatment facilities at tributary mouths to reduce the lake’s overall nutrient inventory over time.

## Introduction

**Fig. 1:**
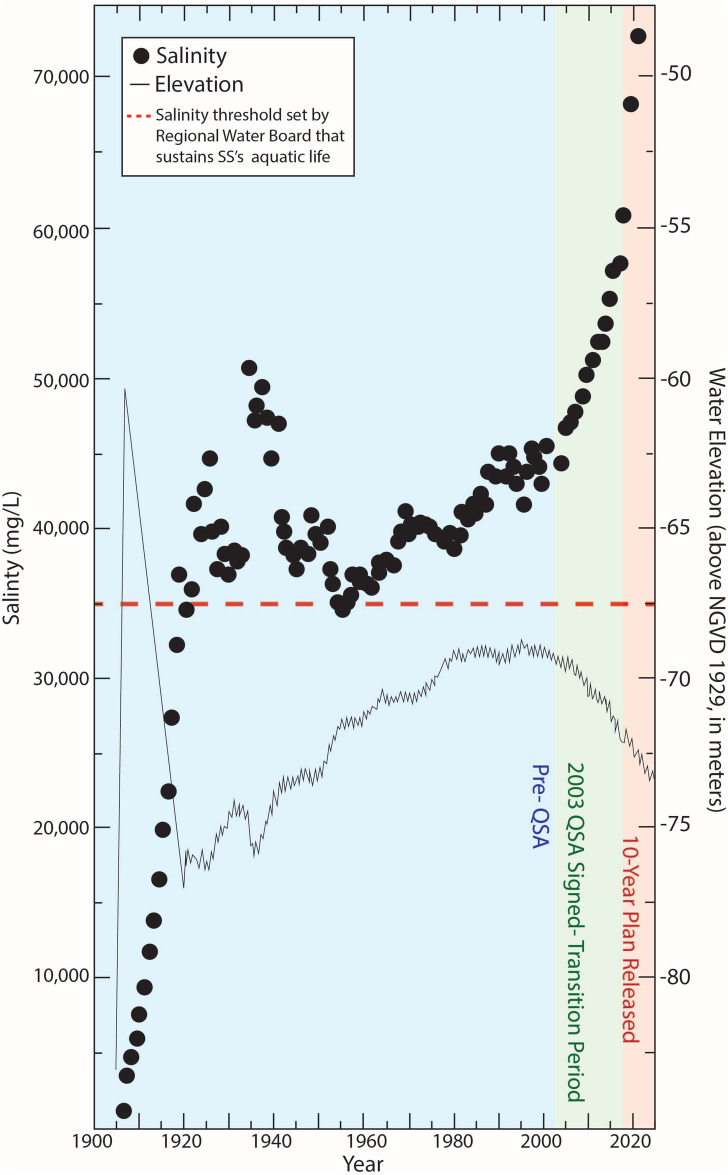
Lake elevation (right y-axis) and salinity (left y-axis) trends from 1905 to 2022 are shown. ‘QSA’ stands for the Quantification Settlement Agreement. The data, obtained from the USGS historical data and Westmorland gauge (ID 10,254,005), are publicly available^7^.

Human activities play an important role in shaping the health of watersheds and the changing geochemistry of the lakes they host^1^. In southern California against a backdrop of ongoing drought, agricultural practices and water usage pose threats to the ecosystem and public health within a growing population^2^. The terminal Salton Sea (SS), California’s largest lake, is sourced from the Colorado River and covers approx. 800 sq. km in the southeastern desert of the Coachella and Imperial valleys^3^. In its current form, the SS reflects the outcome of anthropogenic modifications to the Salton Sink. Prior to recent human overprints, the Colorado River periodically flooded the Salton Sink to form Lake Cahuilla. However, to provide the growing agricultural industry with a consistent water source, settlers diverted water from the Colorado River through a network of canals. In 1905, particularly heavy flow rates of the Colorado River breached irrigation canals, flooding the Salton Sink and creating the current SS^4^. As lake level declined after the initial flooding (Fig. 1), an executive order signed by President Coolidge in 1924 authorized input of agricultural wastewater to the Salton Sink (defined as, “storage of wastes and seepage water from irrigated land”)^5^. The lake has been sustained mainly by untreated agricultural return flow for the last century despite multiple changes in policy regarding water allocation^6^. It receives consistently high loading of nutrients and suspended solids year-round from three main tributaries (the Whitewater, New, and Alamo rivers) and approximately fifty surrounding agricultural canals.

Maximum water depths in the SS, currently at roughly 10 m, are shallow relative to historical levels due to high evaporation rates (simulations show ~ 24% loss of total volume to evaporation per year) and declining riverine input (−16.4 Mm^3^yr^−2^) controlled by state and regional water transfer agreements^8^ (Fig. 1). Water levels have been declining at a rate of 0.3 m/year since 2017 according to readings from the local USGS gauge in Westmorland^7^. Concentrations of total dissolved solids (approx. 65–70 ppt, including calcium, magnesium, potassium, sodium, bicarbonate, chloride, and sulfate) are currently twice those of the Pacific Ocean due to accumulation over the last century as water evaporates in the terminal lake. The 2003 Quantification Settlement Agreement (QSA) was a landmark water-sharing agreement that redefined Colorado River water rights among California water agencies to help the state meet its 4.4 million acre-feet annual allocation limit. The QSA outlined the nation’s largest agricultural-to-urban water transfer, moving up to 200,000 acre-feet annually from Imperial Valley to San Diego County for 75 years. While the QSA secured water rights for Imperial Irrigation District (3.1 million acre-feet) and Coachella Valley Water District (330,000 acre-feet), it also required environmental mitigation for the SS, which received reduced agricultural runoff as a result^9^. The current rapid rate of decrease in water level reflects the termination of mitigation flows of 200,000 acre-feet of water annually to the SS by the Imperial Irrigation District (IID) as of January 2018 per a 15-year delivery requirement as part of the QSA. The mitigation flows aimed to temporarily buffer the lake against reduced agricultural drainage while giving stakeholders time to develop restoration plans and to protect the environment by slowing salinity increases, maintaining water levels, and supporting ecosystem functions during this period. Despite the mitigation period, restoration efforts are slow, and the SS continues to deteriorate with accelerated salinity, persistent eutrophication, ecosystem collapse, and increasing air quality concerns from exposed playa. In addition to these uncertainties, the recent August 2024 IID System Conservation Implementation Agreement (SCIA) will further reduce inflows to the Salton Sea up to 900,000 acre-feet over the next three years.

The 2017 version of the Water Quality Control Plan of the Colorado River Basin RWQCB stated that the primary purpose of the SS is for "collection, transport, and/or storage of drainage (including subsurface) waters from irrigated cropland in order to maintain adequate soil salinity balance for agriculture in the Region." However, the same document also made clear that federal regulations, as per the Clean Water Act, 40 CFR Section 131.10(a), demand that waste transport or assimilation cannot be designated as beneficial uses for any waters of the United States^10^. Despite this inconsistency, this primary function of collecting agricultural return flow^11^remained in place in the 2019 version of the plan as a long-term component of the post-QSA mitigation period^12^. Most authors suggest that external phosphorus loading is the main cause of excessive algae growth and eutrophication in the SS. Schroeder et al. (2002) reported that phosphorus is the limiting nutrient relative to nitrogen, with 26 times more bioavailable nitrogen than phosphorus in the SS^13^. Agricultural surface discharge from the Imperial Valley drained by the Alamo and New Rivers delivers about 80% of the lake’s phosphorus inflow, with two thirds of the rivers’ total phosphorus load as soluble reactive phosphorus (SRP; consisting largely of inorganic orthophosphate [PO_4_]), which is the most bioavailable fraction for algae growth. The remaining third is present as particulate phosphorus.

The SS’s rapid water level decline since 2003 has fundamentally altered its ecosystem, rendering baseline studies from the early 2000s outdated^13,14^. This paper examines two decades of evidence documenting persistent nutrient pollution and its ecological impacts at the SS^15^, spanning the period before and after the 2003 QSA’s mitigatory measures^16–18^. Despite the critical implications of nutrient contamination and hyper-eutrophication at the SS for public and environmental health^19^, this issue remains notably absent from both the Salton Sea Management Program’s 10-year plan^20^and its Long-Range Plan^21^. We raise nutrient dynamics as central to the lake’s deteriorating water quality and essential for developing effective management solutions in coming decades^22,23^. Through documenting external nutrient inputs, seasonal cycling patterns, and their impacts on the aquatic environment, we provide a comprehensive overview of the current state of nutrient pollution. While we touch on potential remediation strategies, our primary aim is to establish a foundation for future research—not only for the Salton Sea but also for other eutrophic water bodies. This work highlights how water quality challenges intersect with broader issues of water supply management, agricultural practices, and national food security within the context of climate change and increasingly modified environments of the twenty-first century^24–26^.

## Results

### Lake inflow and salinity

The historical trends for lake elevation and salinity in the SS from 1905 to 2022 (Fig. 1) indicate that elevation peaked shortly after the lake’s creation in 1905. A decade later, levels rapidly declined until 1924 when an Executive Order permitted agricultural discharge to the lake, leading to a sustained rise until the 1990s^27^. Salinity initially peaked following inputs of agricultural return flow, but increasing flows lowered the salinity to a threshold suitable for aquatic life (i.e. 37,000 mg/L or 37 ppt^12^;). With the slowing of lake level increase by the 1980s, however, evaporation rates began to exceed inflow, leading to rapid salinity increase and lake level decrease. After the implementation of the QSA in 2003 and phasing out of delivery of mitigation water in 2018, SS lake levels declined rapidly as water inflow decreased— on the order of 200,000 acre-feet per year transfer to San Diego and other areas^28^. Meanwhile, salinity has increased consistently (Fig. 1) due to the evaporative nature of the terminal lake^17^.

### External nutrients source and loading

**Fig. 2:**
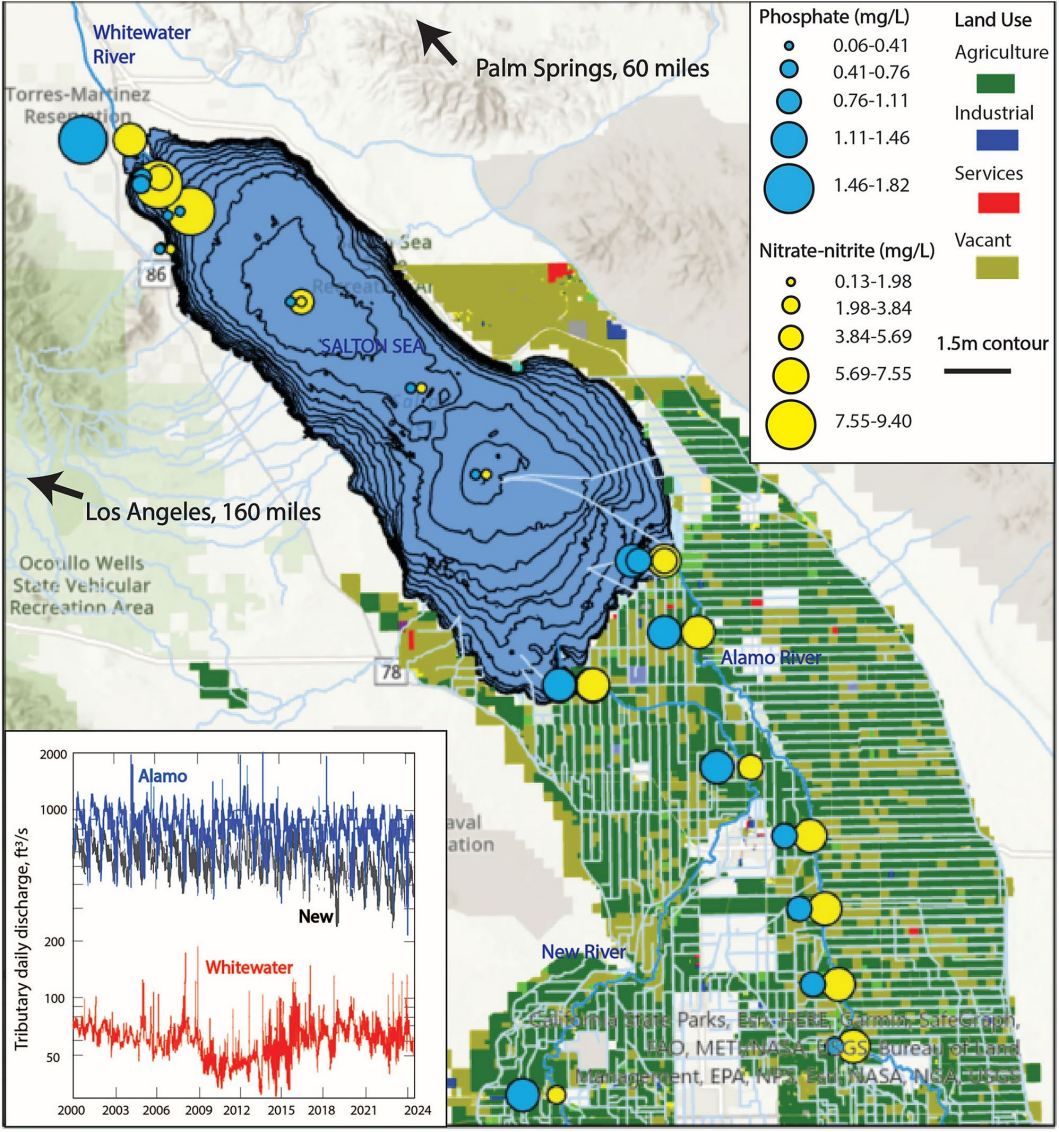
Map of the Salton Sea and its tributaries (Whitewater, Alamo, and New Rivers), displaying bathymetric contour at 1.5 m increments within the lake produced by the Bureau of Reclamation in 1999, and Imperial County land using data compiled by Southern California Association of Governments in 2016^31^. Total phosphate and nitrate-nitrite data in the lake and along the Alamo and New Rivers were collected on March 19, 2019 and distributed by CEDEN, IID and Bureau of Reclamation. Nutrients data at the Whitewater river mouth were collected by Salton Sea Environmental Time Series on December 3, 2022, accessible at https://saltonseascience.org/^32^. Tributary daily discharge (ft^3^/m) are shown for Alamo, New and Whitewater rivers (source: USGS NWIS^33,34^) from year 2000 to 2024. Vacant land (light green) represents undeveloped areas that are neither in agricultural use, designated as open space/recreation, nor protected/undevelopable land, based on SCAG 2016 Land Use Codes. This designation is independent of seasonal changes and includes subcategories of undifferentiated vacant land, abandoned agricultural land, and areas with limited improvements. Desert areas not labeled as vacant may fall under other categories such as wildlife preserves, open space, or protected land.

The spatial distribution of nutrients in the SS ecosystem is largely due to agricultural land use in the surrounding areas. Figure 2shows the locations of the three main tributaries, Alamo, New, and Whitewater rivers. These inflows release phosphate and nitrate in hypereutrophic concentrations from agricultural runoff (conservative literature values for hyper-eutrophication thresholds for nitrate and phosphate concentrations are 1.5 mg/L and 0.05 mg/L, respectively^29,30^). Data points in 2019 and 2022 were mapped together as a representation of the most recent data for surface nutrient concentrations available in both the north and south. Although surface nutrients are concentrated at the mouth of the SS tributaries, they are reduced in concentration in the interior of the SS as they are taken up by algae and circulated within the water column.

**Fig. 3:**
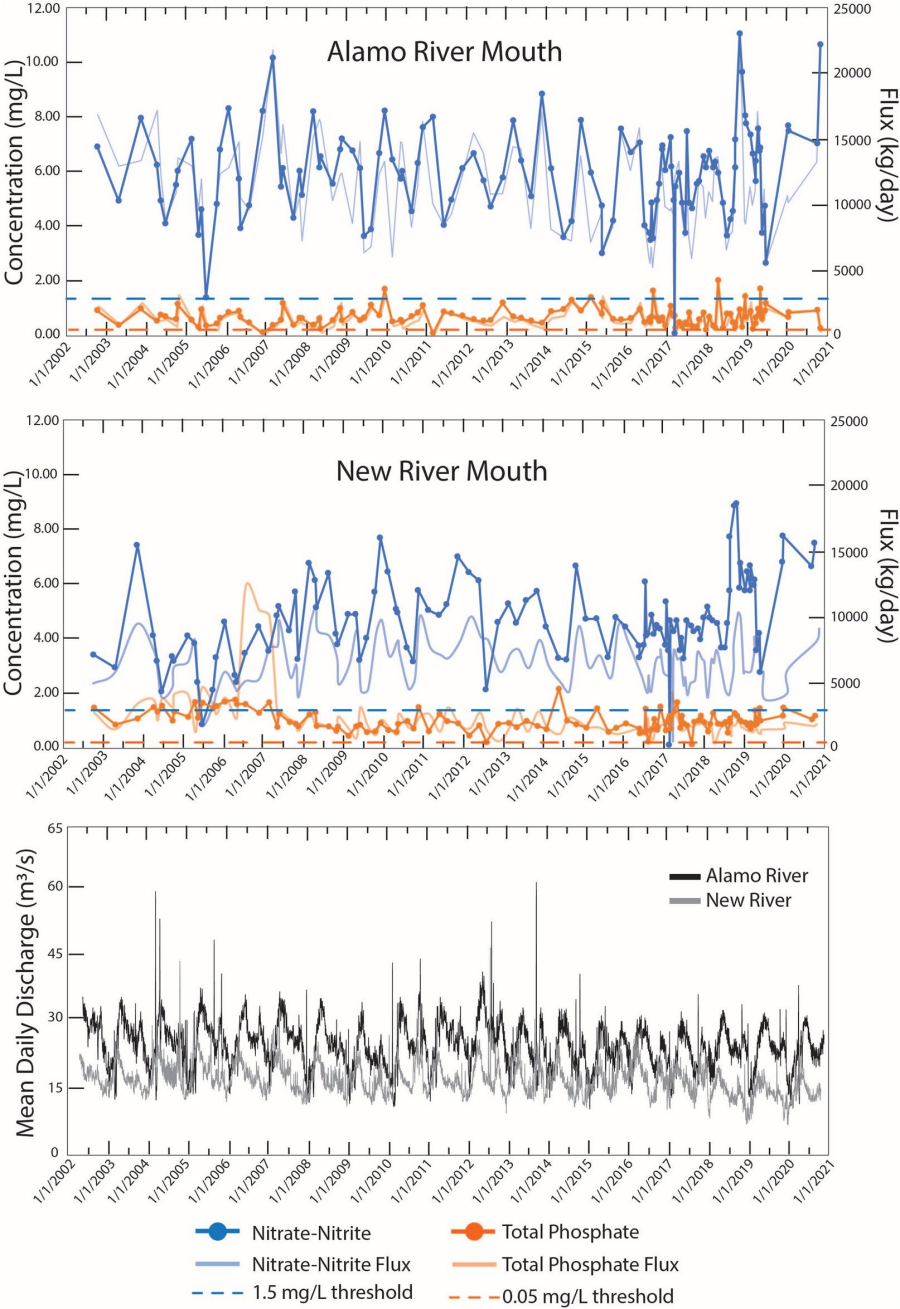
Total concentration and fluxes of nitrate-nitrate and total phosphate at the mouths (signifies loading) of the New and Alamo Rivers from 2002 to 2020, as well as the mean daily discharge (cubic meter per second) of the New River (ID 10,255,550^33^) and Alamo (ID10254730^34^). The fluxes were calculated from the average monthly discharge rates. Dotted lines show literature value of general hypereutrophication thresholds for nitrate-nitrite and phosphate concentrations^25,26^. The data were compiled and provided by Michael Stenstrom and Meng-Chen Lee at UCLA from personal collections and publicly available sources from Bureau of Reclamation^35^, IID^36^, and CEDEN^37^.

Nutrient concentrations and fluxes at the southern tributary outlets as well as the flow rates of the Alamo and New Rivers are shown in Fig. 3. Both Alamo and New Rivers in the south have daily discharge about ten times higher than that of the Whitewater River in the north (Fig. 2), which suggests that the total flux of nutrients into the southern end of the lake are significantly higher. Because of this southern bias in inputs, as well as limitations in data availability for the north, focus in this study in placed on the nutrient loading flux to the SS via the southern tributaries. Input concentrations consistently exceed literature definitions of hyper-eutrophication, although the EPA’s TMDL (Total Maximum Daily Load) standards for nutrients vary depending on the water body and its regional characteristics. Nitrate concentrations in the mouths of the Alamo and New River tributaries were observed to be higher during the winter to spring compared to the summer to fall, even though levels of discharge were higher during the spring, suggesting that dilution was not the primary control (Fig. 3). Phosphate concentrations at tributary mouths were found to be more consistent across seasons but fluctuated interannually. The tributary discharge exhibited a distinct semi-annual seasonality pattern, with the highest flow occurring in spring reflecting regional irrigation practices, gradually decreasing through late summer, followed by a second smaller pulse from the fall monsoon season before winter, the driest season.

### Seasonal nutrients cycling

**Fig. 4:**
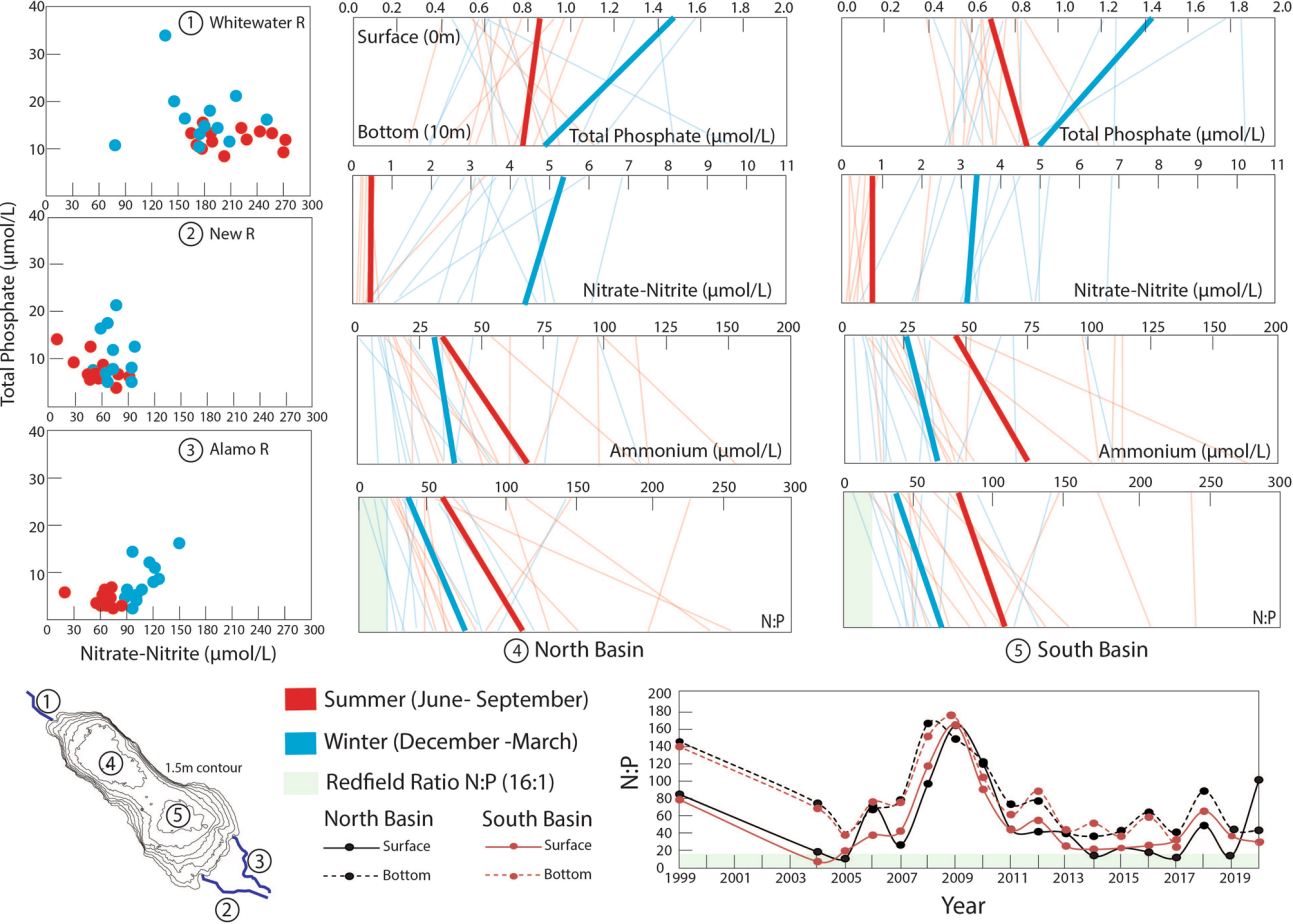
Seasonality of nutrients cycling are shown through: panels 1–3 for nitrate-nitrite (x-axis) and total phosphate (y-axis) concentrations of the Whitewater, New, and Alamo Rivers gathered from independent sampling events from 2004 to 2017; and panels 4–5 for total phosphate, nitrate-nitrite (NO_3_, NO_2_), ammonium (NH_3_) concentrations and the N:P (x-axis) in surface (0 m) and deep (10 m) (y-axis) of the water column in the deepest locale in the north and south basin from 2004 to 2017. The bottom panel shows N:P trends throughout time from 1999 to 2019 in the same locations in the north and south basin. Data from the winter (categorized as sampling dates falling in between 12/21-3/21) are depicted as blue and those from the summer (6/21-9/21) are depicted as red. In panels 4–5, actual concentrations from independent sampling events are shown in lightened lines whereas averages taken from the actual concentrations are shown in bold lines for respective winter and summer periods. Data for the Salton Sea tributaries and water column were gathered from sampling events spanning 2004 to 2017 and are publicly available from the Bureau of Reclamation^35^. Tributary samples were collected at the Whitewater River at 33.52482, −116.07894 (Lincoln St. intersection). The New River was accessed at 33.08548, −115.61451 (Gentry Rd. intersection), while Alamo River was accessed at 33.19924, −115.59710 (along Red Hill Rd. near the Red Hill Marina). The northern and southern deepest locale of the lake was accessed at 33.40013, −115.92574 and 33.26265, −115.739, respectively.

Figure4presents tributary and water column (surface 0 m vs. bottom 10 m) nutrient concentrations from 2004 to 2017, showing both individual measurements (dots/lighter lines) and averages (bold lines). Tributary mouth measurements, taken upstream of lake water mixing, reveal higher nutrient concentrations during winter compared to summer. The Whitewater River consistently shows the highest concentrations (> 1 mg/L) of total phosphate and nitrate-nitrite among the three major tributaries in both seasons. While the Alamo and New rivers show overlapping seasonal ranges for both nutrients, the Whitewater River exhibits distinct concentration ranges.

Water column measurements from the deepest locations in north and south basins show comparable nutrient patterns. Total phosphate exhibits strong seasonal variation in surface waters (lower in summer than winter) but only minimal seasonal changes in bottom waters. Nitrate-nitrite concentrations are significantly lower in summer throughout the water column, with similar surface and bottom concentrations in both seasons. Ammonium, measured only in the water column due to negligible levels in oxic tributaries, shows elevation during summer, particularly in frequently anoxic bottom waters. These distributions reflect complex cycling between distinct sources and sinks, including external nonpoint-source inputs^38^, internal release through microbial decomposition, redox-dependent processes, and sediment resuspension^39,40^. N:P molar ratios consistently exceed the Redfield ratio (16:1) in both basins, particularly during summer (> 50:1), though some winter samples fell below 16:1. Statistical analysis using probability density functions (establishing exponential distribution) confirms this pattern: N:P ratios exceed the Redfield ratio with 0.99 probability in summer (mean ± SD = 84 ± 64) and 0.99 probability in winter (mean ± SD = 57 ± 45), demonstrating persistent deviation from Redfield stoichiometry throughout the year. Time-monitored plot of N:P ratios from 1999 to 2019 reveals consistent spatial patterns between north and south basins, with bottom waters showing notably higher ratios (80:1 to 100:1) compared to surface waters (40:1 to 60:1). This vertical gradient likely reflects elevated ammonium concentrations in bottom waters. A pronounced peak in N:P ratios occurred between 2007–2009, followed by a return to baseline levels in 2011. While this peak does not correspond to documented changes in external nutrient loading, it may reflect shifts in internal nutrient cycling mechanisms, highlighting the need for further investigation into interannual variations in the lake’s biogeochemistry.

### Dissolved oxygen

**Fig. 5:**
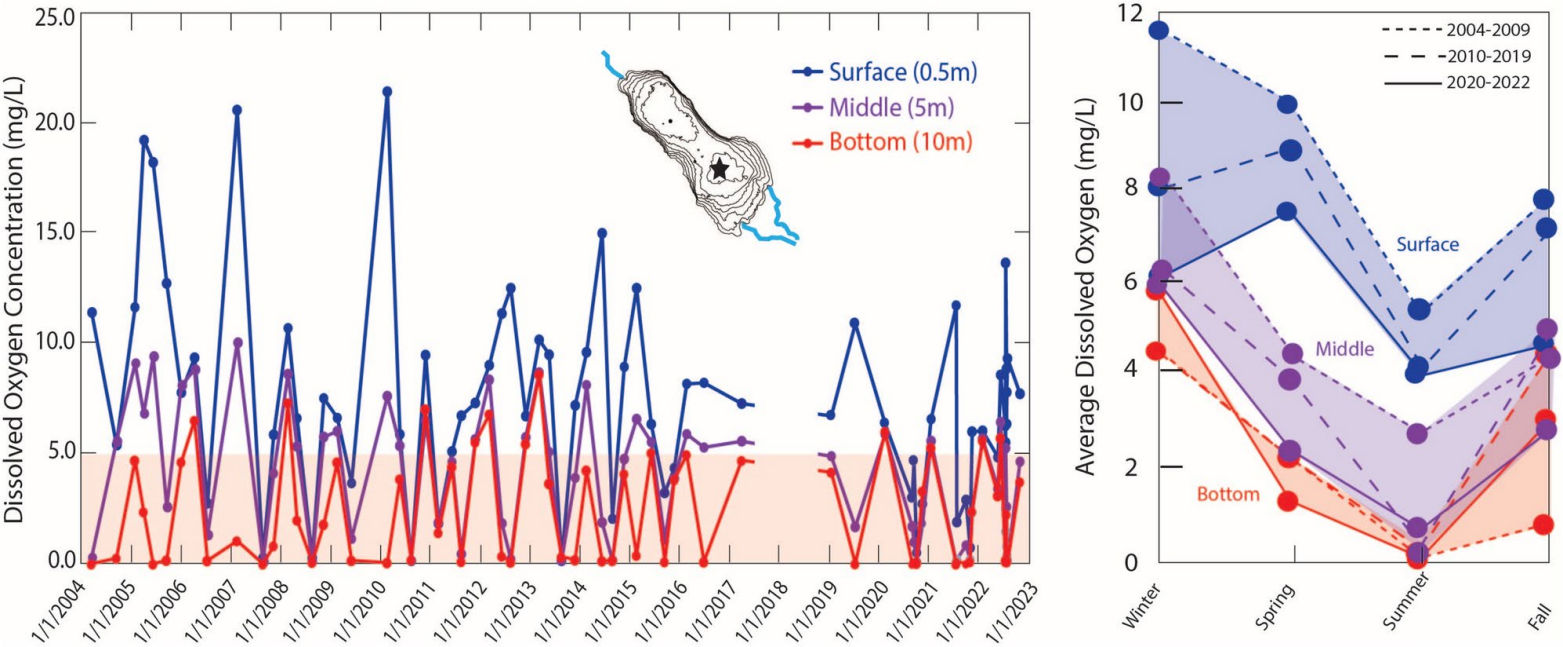
Dissolved oxygen levels in the Salton Sea (2004–2022; left) show declining water quality due to consistent nutrient loading and salts accumulation where red tinted region shows the hypoxia threshold (< 5 mg/L)^11^. Samples were taken periodically at three depths (surface 0.5 m, middle 5 m, and bottom 10 m) at the southern deepest locale of the lake (indicated by the star symbol in the left figure subset; 33.26265, −115.739). Data were collected by the Bureau of Reclamation (2004–2020)^35^and Lyons lab (2020–2022). There is a gap in 2018 because of missing data for most of that year^35^. Averages were taken on the same data for each season (Winter, Spring, Summer, and Fall; right) to indicate changes in dissolved oxygen availability throughout the periods 2004–2009 (tight dashed line), 2010–2019 (dashed line), and 2020–2022 (full line). For numerical averages and standard deviations, see Table 1.

Dissolved oxygen concentrations at the SS’s deepest point in the southern basin show significant depth-dependent variations across seasons and a long-term declining trend from 2004–2022 based on averages, although standard deviations show a wide spread (Fig. 5; Table 1). The data reveal complex temporal patterns that are not well-characterized by simple linear regression, as dissolved oxygen often drops to 0 mg/L, particularly in deeper waters. Linear regressions for surface, middle, and bottom waters show slightly negative slopes (< −0.0005) with weak correlations (R-squared values of 0.0698, 0.1345, and 0.0051, respectively). To better understand temporal changes following the 2003 Quantification Settlement Agreement (QSA), we analyzed the data in three time periods: 2004–2009, 2010–2019, and 2020–2022. We analyzed decadal-scale changes using seasonal averages and standard deviations across three time periods, with total measurements (sample size) of 18, 29, and 22 respectively. While most seasons had at least 4 measurements per period, winter sampling in 2020–2022 was limited to 2 measurements, which should be considered when interpreting seasonal patterns. This analysis revealed consistent seasonal cycling, with highest dissolved oxygen in winter and lowest in summer, alongside a decline in surface water oxygen levels, with winter averages decreasing from 11.5 mg/L to 6.2 mg/L and similar decreasing trends for all seasons over the study period. However, winter and fall bottom waters show an increasing trend, noticeably from 0.8 mg/L to 3.0 mg/L in the fall. Keep in mind that Table 1 shows the wide spread in standard deviations and uneven sample sizes due to limitations in data availability. Both USBR (pre-2020) and the Lyons lab (post-2020) followed standard protocols for dissolved oxygen measurements using a sonde, and the observed oxygen decline and increase in surface and bottom waters, respectively, precedes the transition between monitoring programs, suggesting a trajectory that is real rather than being an artifact linked to differing measurement or calibration protocols.

**Table 1:** Averages, standard deviations, and sample sizes (#) for dissolved oxygen content (mg/L) throughout the water column in assorted time periods to accompany Fig. 5.

Water column depth	Period	Winter			Spring			Summer			Fall		
		Average	St. Dev.	#	Average	St. Dev.	#	Average	St. Dev.	#	Average	St. Dev.	#
Surface (0.5 m)	2004–2009	**11.5**	5.5	5	**10.1**	5.9	5	**5.4**	8.6	4	**7.9**	3.4	4
	2010–2019	**8.2**	3.1	9	**9.0**	3.3	8	**4.1**	4.8	6	**7.3**	1.8	6
	2020—2022	**6.2**	0.2	2	**7.6**	3.4	10	**4.1**	3.3	5	**4.8**	2.9	5
Middle (5 m)	2004–2009	**8.4**	1.5	5	**4.5**	3.7	5	**2.7**	4.5	4	**4.5**	1.5	4
	2010–2019	**6.2**	2.3	9	**3.9**	1.8	8	**0.4**	0.4	6	**5.1**	1.1	6
	2020—2022	**5.8**	0.1	2	**2.3**	2.2	10	**0.8**	0.7	5	**2.9**	2.2	5
Bottom (10 m)	2004–2009	**4.5**	2.2	5	**2.2**	2.6	5	**0.1**	0.1	4	**0.8**	0.7	4
	2010–2019	**4.4**	2.6	9	**2.2**	2.2	8	**0.2**	0.1	6	**4.4**	2.3	6
	2020—2022	**5.8**	0.3	2	**1.2**	1.9	10	**0.0**	0.0	5	**3.0**	1.9	5

## Discussion

As the “agricultural sump” of the Imperial Valley, the SS has accumulated a century’s worth of agricultural discharge containing nutrients, organic matter, salts, and non-biodegradable pollutants^41^. While the process of eutrophication—the accumulation of nutrients and organic biomass—is natural and occurs in virtually all bodies of water^42^, anthropogenically accelerated scenarios, known as cultural eutrophication, have deteriorated the water quality in phase with decreasing water depth due to evaporation and reduced inflow^43,44^. The historical trends for lake elevation and salinity in the SS provide insights into the lake’s changing environmental conditions. The initial rise in lake elevation after its creation in 1905 was followed by a rapid decrease, which was reversed when irrigated agriculture expanded in the valley. The sustained increase in lake levels until the 1990s provided opportunities for the development of various water-dependent ecosystems^45^. However, the gradual decline in lake levels after the QSA in 2003 and rapid decline starting in 2018 have had significant impacts on the lake’s ecological and socio-economic sustainability. The concurrent increase in salinity (10,000 mg/L, or 10 ppt, over five years since 2018) through tributary loading and agricultural drainage in combination with evaporation elevated the threats to various salinity-sensitive aquatic species. The SS has not been nor is it currently in steady state, defined as the condition where inputs and outputs of a particular substance are equal so that concentrations in the reservoir remain constant. The lake has been experiencing changes in water levels, salinity, and nutrient concentrations since its creation due to changing water inflows, evaporation, and nutrient loading. These fluctuations have added instability to the ecosystem^17^.

### External nutrient loading

External nutrient loading at the SS occurs primarily through three main tributaries and surrounding agricultural drains. While the Whitewater River shows the highest surface nutrient concentrations, the southern tributaries (Alamo and New Rivers) contribute the majority of nutrients due to approximately ten times higher daily discharges (Fig. 2). Despite this spatial variation in loading, the lake remains well mixed, with northern and southern basins showing similar nutrient concentrations and redox patterns (Fig. 4; panel 4 and 5). Average annual loading estimations from the southern tributaries demonstrate substantial nutrient inputs: the Alamo River contributes 4,110,191 kg of nitrate-nitrite and 520,750 kg of total phosphate, while the New River adds 2,197,959 kg of nitrate-nitrite and 631,317 kg of total phosphate. While a complete nutrient mass balance would be useful, it requires additional data on Whitewater River contributions, agricultural canal inputs, internal nutrient cycling, and permanent removal processes like denitrification, organic matter burial, and inorganic P mineralization, which are beyond the scope of current work but are plans for near-future research.

Seasonal patterns in nutrient loading reflect interactions among agricultural practices, hydrology, and biological processes (Fig. 3). During winter and spring, the Alamo and New Rivers show higher discharge rates due to pre-planting soil leaching practices. Unlike typical systems where increased water volume dilutes nutrients^46^, these tributaries maintain elevated nutrient concentrations, driven by spring fertilizer applications and reduced biological uptake in cooler waters. This combination of high discharge and elevated concentrations results in maximum nutrient delivery to the lake, leading to significant accumulation. During summer, the lake’s nutrient dynamics undergo a notable shift as tributary inputs decline. Field observations reveal active biological processes in the tributaries, particularly the Alamo River, where algal blooms and oxygen supersaturation suggest significant nutrient uptake even during the brief journey from agricultural areas to the lake. Despite this reduction in external nutrient delivery, summer algal blooms in the lake persist due to a complex interplay of factors—elevated temperatures, accumulated nutrients from spring runoff, and internal phosphorus released from lake sediments—creating ideal conditions for excess algal growth.

### Seasonal nutrients cycles and redox variation

**Fig. 6:**
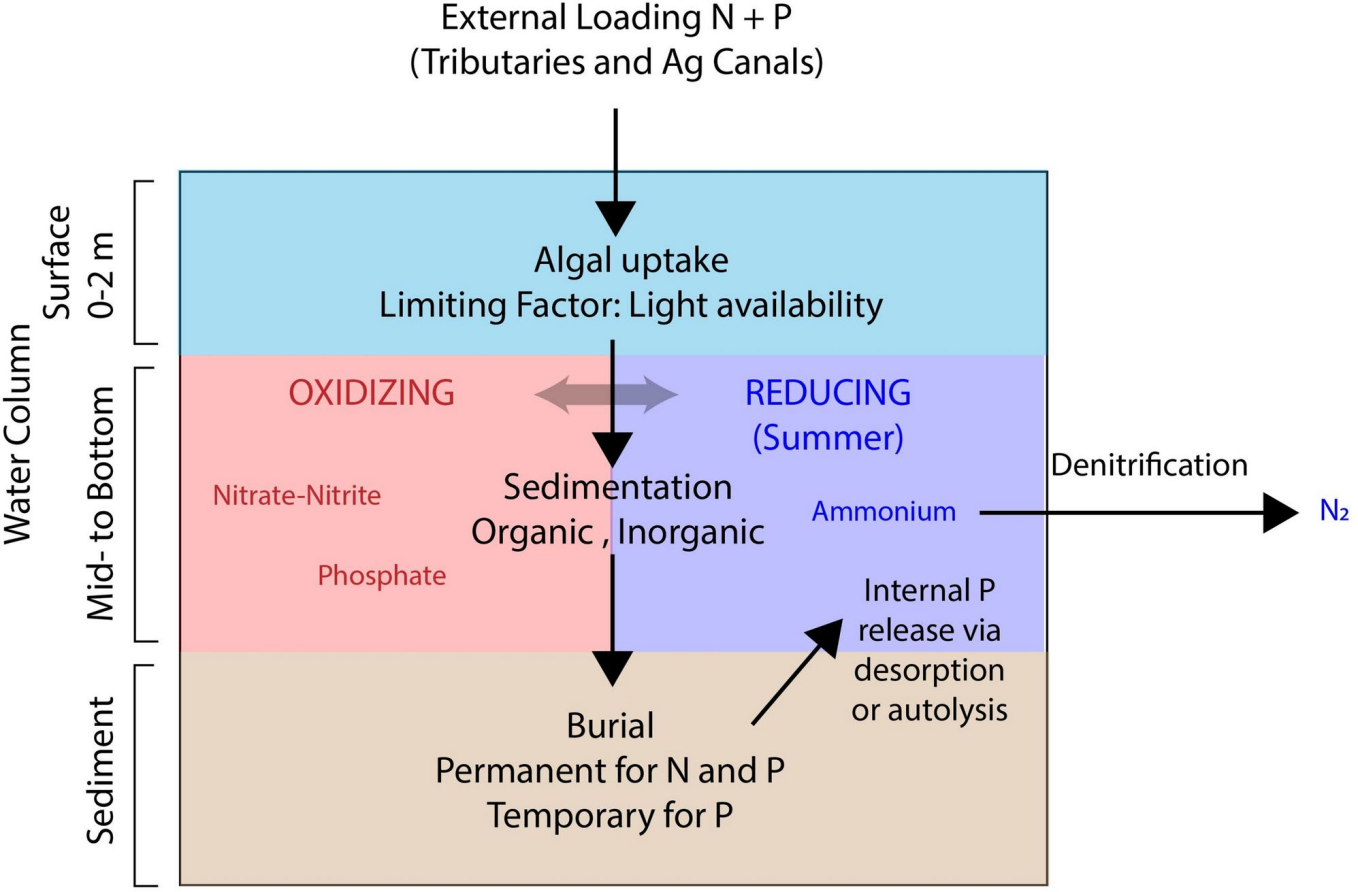
Conceptual diagram illustrating seasonal nutrient cycling in the Salton Sea. Key processes include: algal uptake and primary production, organic matter decomposition, sediment–water nutrient exchange, redox-dependent transformations, and permanent and temporary sediment removal pathways.

Redox conditions in the SS drive seasonal and spatial patterns of nutrient transport between the water column and sediments^47^. As illustrated in Fig. 6, these biogeochemical processes vary seasonally, with summer conditions characterized by high temperatures (~ 35 °C) and prolonged light exposure, leading to enhanced algal growth and microbial activity. These conditions create temperature-dependent stratification and oxygen consumption, resulting in low oxygenated or anoxic bottom waters.

Despite evidence for thermal water column stratification during the summer, conditions then show uniform phosphate concentrations (~ 0.08 mg/L) throughout the water column, reflecting a balance between high biological uptake and vertical mixing from diurnal stratification collapse^48^ (Fig. 4, panel 4–5). In contrast, well-mixed, oxygenated waters during the winter develop a distinct nutrient cycling pattern: surface waters maintain higher phosphate concentrations (0.14 mg/L), while bottom waters show lower concentrations (0.09 mg/L). Oxygenated water favors phosphate sequestration in the bottom water during winter. During summer reducing conditions, phosphorus stored in sediments can remobilize through desorption and be released internally. The release of soluble reactive phosphorus (SRP) into surface waters requires mixing events to transport it through the water column. Historically, the lake experienced predictable mixing during late summer monsoons and the fall transition period. As the lake has become shallower, its thermal stratification has weakened, leading to more frequent mixing events that can transport sediment-derived SRP to surface waters where it becomes available for biological uptake^48^. While site-specific measurements of diffusive P fluxes at the sediment–water interface would be needed to make quantitative comparisons to external P loading rates, important parallels for the SS’s internal phosphorus cycling can be drawn to the Baltic Sea system as documented by Mort et al. (2010)^49^. There, seasonally hypoxic sites show significant sediment–water PO_4_ exchange (180–440 μmol P m⁻^2^ d⁻^1^, up to 800 μmol P m⁻^2^ d⁻^1^) during hypoxic periods. The SS likely experiences similar or higher internal loading rates given its hypereutrophic state and higher temperatures. However, key differences exist: while the Baltic Sea’s internal loading derives from both Fe-oxide bound P and organic P (the latter comprises 60–80% of total reactive P burial in Baltic sediments), the SS’s iron-limited, sulfide-rich sediments rely primarily on organic P. Under anoxic conditions, P release intensifies through two mechanisms. First, high sulfide conditions bind iron in iron sulfides (FeS_2_), preventing P capture or releasing it via Fe reduction. Second, microbial stress response during anoxia preferentially releases P from organic matter, as shown by increasing C_org_/P_org_ratios (140 under oxic relative to 1500 under anoxic conditions)^49^. The magnitude of this preferential P release would be significant in summer when anoxic conditions develop. This is especially important because Mort et al. found no evidence for significant transformation to more stable P forms (like authigenic Ca-P) in anoxic basins, suggesting that P released from organic matter returns directly to the water column, creating a positive feedback loop that maintains eutrophic conditions. These relationships suggest that even if external loading were reduced, internal loading from decomposing organic matter could sustain poor water quality for extended periods.

Seasonal redox shifts also drive nitrogen cycling through multiple pathways^50^. While a portion of nitrogen is permanently removed from the system through denitrification to N_2 _gas and organic matter burial in sediments, much of the nitrogen pool remains actively cycled within the lake, transforming between nitrate, ammonium, and organic forms based on seasonal redox conditions and biological uptake^51,52^. This internal cycling maintains high N:P ratios and supports continued productivity: summer surface waters show reduced nitrate-nitrite concentrations (~ 0.05 mg/L) due to high biological uptake. Winter conditions maintain higher nitrate-nitrite levels throughout the water column (~ 0.2–0.3 mg/L) due to reduced biological uptake and inhibited denitrification in oxygenated waters. Ammonium shows distinct seasonal patterns, with high concentrations (~ 1.5 mg/L) beneath the chemocline in reducing conditions from organic matter decomposition in summer, and lower concentrations (~ 0.5 mg/L) mainly from fertilizer inputs and organic matter breakdown in winter. The complex interplay of these nutrient cycling processes is summarized in Fig. 6, which illustrates the seasonal variations in phosphorus and nitrogen transformations^53^, including internal loading mechanisms, redox-dependent processes, and the coupling between water column and sediment dynamics. These processes collectively maintain the lake’s hypereutrophic state through feedback loops that persist even as external conditions change.

### Other limiting factors for primary production

Prior studies^13,15,54^have argued for phosphate limitation in the SS due to persistently elevated N:P ratios in the waters significantly exceeding the Redfield ratio (> 16:1), with some values exceeding 50:1 and even 180:1. Specifically, Schroeder et al. (2002)^13^ hypothesized that P was being effectively scavenged and irreversibly sequestered in sediments through mechanisms such as apatite formation from fish die-offs and sorption to calcite. However, our current analysis suggests changing conditions in the SS have altered these dynamics. While elevated N:P ratios still persist (Fig. 4), such that P is the limiting nutrient in a stoichiometric sense, they do not necessarily imply phosphorus limitation of photosynthetic activity. Phosphate concentrations consistently exceed eutrophic thresholds (> 0.05 mg/L or 0.53 µmol/L), even during summer (Fig. 4). A one-tailed Student’s t-test confirms these concentrations are significantly above the eutrophic threshold (p = 1.39 × 10⁻⁶). These persistently high phosphate levels are maintained through both high tributary inputs and internal release from sediments during reducing conditions in the summer, creating a self-reinforcing feedback loop that makes phosphorus limitation unlikely under current conditions. Although bi-monthly sampling has temporal resolution limitations, the consistently high P concentrations, combined with observed seasonal patterns of bottom water anoxia and P release, show that internal loading prevents P limitation in the current system.

Current lake conditions differ markedly from those reported by Schroeder et al.^13^. The lake is now shallower with more frequent mixing events, which have fundamentally changed sediment–water interactions and sediment resuspension patterns. Moreover, while Schroeder et al. did not identify internal phosphorus loading from anoxic sediments as significant, our findings indicate it is a considerable source of phosphorus to the lake. Other previous research has highlighted the significant role of internal phosphorus loading in driving eutrophication, with sediment resuspension via wind-induced wave activity and associated organic decomposition as the primary mechanism for nutrient cycling^14,15^. Sediment desorption has also been identified as a critical factor in orthophosphate loading within the water column^55,56^. This previously published calibrated one-dimensional hydrodynamics/water quality numerical model (DLM-WQ) suggested that nutrient limitations primarily affect phytoplankton growth near the surface, while light availability limits growth below 3 m. The simulation demonstrated that reducing external tributary phosphorus loads by 90% had minimal short-term effects on chlorophyll a concentration and phytoplankton growth, highlighting the importance of internal loading from sediment resuspension in the nutrient budget^40^. Further, Lee^55,56^suggested building artificial islands in the SS to reduce bottom shear stress, thereby enhancing sediment burial activity and ultimately reducing orthophosphate concentrations in the water column in the long-term, although the rate of this process remains uncertain given limited sediment data in the SS. Other limitations on primary productivity, despite the eutrophic conditions, may arise from diurnal stratification collapse (especially with more mixing due to the decreasing depth of the water column) and light availability limiting growth (modeled in^57^; as seen in eutrophic Lake Victoria [Uganda] and Lake Biwa [Japan]^58,59^, respectively]). Additional controlling factors include alterations in food web structure (microbial competition and lack of higher trophic levels^60^) and dissolved oxygen saturation and pH in surface waters^61^. Only by sampling these variables at high resolution will we be able to capture in detail their spatial and temporal heterogeneities and full range of controls on productivity in the SS^61^.

### Ecological effects related to oxygen availability

Our data suggest an overall decline in dissolved oxygen levels at the SS, despite high variability from surface water supersaturation during periods of intense primary productivity. This decline reflects the interplay of multiple stressors: eutrophication, increased salinity, and high seasonal temperatures^62^. While algal photosynthesis temporarily increases surface oxygen levels, subsequent decomposition and respiration deplete oxygen, particularly in deeper waters. Higher salinity and temperature further compound this problem by reducing the water’s capacity to hold oxygen^63^. Recent changes in water column dynamics (Fig. 5) suggest a likely shift in oxygen distribution patterns. As lake depth decreases, the previously stable temperature-dependent stratification has been disrupted, leading to more frequent occurrence of mixing events throughout the water column. Instead of maintaining the distinct anoxic bottom layer characteristic of the early 2000s, we now observe lower oxygen conditions throughout the entire water column. More frequent mixing could lead to lower surface dissolved oxygen because poorly oxygenated bottom waters are mixed upwards more frequently. At the same time, this mixing results in oxygenation of anoxic waters in the bottom. Further research is needed to characterize the thermal and chemical stratification patterns of this new regime, particularly how reduced water depth affects mixing dynamics and biogeochemical cycling. This altered oxygen regime may have severe ecological implications. In the past, when the lake was deeper and more stably stratified during summer, episodic wind events caused mixing of bottom waters into oxygenated surface layers. These events could be catastrophic—for example, one such mixing event in August 1999 resulted in 7.9 million fish deaths in a single day^64^. However, the current state of persistent low-oxygen conditions throughout the water column may be equally detrimental to long-term ecosystem health^65^, as it prevents the community recovery that was possible between historical episodic events.

Cultural eutrophication has fundamentally disturbed trophic relationships through multiple mechanisms. Harmful algal blooms (HABs), occurring regularly during warm months, produce toxins that threaten higher trophic levels in the SS^66^. Historical data shows 85% of water samples contained detectable microcystin levels between 1999–2001, primarily from Synechococcus and Oscillatoria cyanobacteria^67^. These blooms impact wildlife through both direct toxicity and oxygen depletion following bloom die-offs^66,68,69^, contributing to dramatic loss of resident fish populations^70^. The oxygen-depleted conditions create additional environmental hazards. Anoxic bottom waters and sediments sequester toxic heavy metals^41,63,71–73^, which could be remobilized as lake levels drop and lake floor is exposed, leading to transportation as playa dust, threatening nearby communities^74–76^. Moreover, high sulfate and organic availability enables microbial sulfate reduction under anoxic conditions, producing hydrogen sulfide^77–79^. Wind transport can carry this sulfide to neighboring communities and the greater Los Angeles region^80^. Current management approaches through the SSMP’s 10-Year Plan^20^focus primarily on habitat construction around the lake. While long-range concepts consider whole-lake restoration, they emphasize salinity control through flow-through systems^21^ without adequately addressing hyper-eutrophication impacts on water quality. Effective ecosystem recovery requires comprehensive strategies that address both nutrient pollution and persistent oxygen loss alongside salinity management.

### Potential solutions with nutrient management

Managing nutrient pollution in the SS requires addressing both legacy phosphorus and ongoing inputs. Empirical modeling^55,56^demonstrates that even a 50% decrease in external phosphorus loading would only reduce near-surface total phosphorus by 25–50%, while achieving moderately eutrophic conditions would require a 70–90% reduction. Given these substantial existing nutrient reservoirs, effective management likely requires combining external load reduction with in-lake remediation strategies. While Lee and Stenstrom^55,56^demonstrated through Delft3D modeling that artificial islands to obstruct water column mixing could potentially lower orthophosphate concentrations through long-term sediment burial, the timeline and overall impact of such interventions remain uncertain and would require bottom waters to stay aerated, which is the current dilemma with eutrophication. Other management approaches include constructed wetlands for reducing new inputs^22,81,82^, though the significantnutrient inventory already present suggests complementary in-basin interventions, such as algae farming, may be essential. Additional options include chemical treatments like aluminum sulfate addition^83^, though this could exacerbate already high sulfate levels from agricultural runoff, or sediment dredging^84^ for physical removal of the top organic layer of sediments.

The SS’s complex nutrient dynamics, where neither nitrate nor phosphorus limit primary production^56^, further support the need for multiple management approaches. While the Regional Waterboard has established TMDLs for sedimentation/siltation in the Alamo and New Rivers^85^, the numeric targets (200 mg/L total suspended solids) are not directly tied to nutrient concentrations, making it difficult to quantify impacts on nutrient loads. Similarly, while proposed dissolved oxygen standards of 5.0 mg/L align with warm freshwater habitat requirements^10^, meeting these targets requires addressing multiple controlling factors as outlined beyond nutrient supply. The current SSMP near-term management focus on dust suppression and habitat creation^20^around the lake, but for long-term plans, should incorporate comprehensive water quality solutions that combine external load reduction through constructed wetlands with in-lake nutrient removal strategies. However, crucial questions remain about implementation, including wetland design specifications, nutrient reduction efficiency, ecosystem services, costs, water consumption, and impacts on lake size and dust emissions. Such technical and economic analyses should be conducted alongside consideration of agricultural policy measures^86–88^ to develop effective, long-term solutions.

## Conclusion

For more than a century, California’s largest lake has been used primarily for collecting untreated agricultural return flow. This study sheds light on seasonal nutrient loading and distribution patterns in the SS. These patterns are characterized by pronounced increases in nutrient loading during the winter and spring, potentially attributed to increased fertilizer application and reduced biological uptake during these seasons, which override the dilution effect resulting from higher tributary discharge during the same periods. Seasonal patterns in nutrient distributions throughout the water column are prominent. In winter, total phosphates and nitrate-nitrite levels are higher due to reduced primary productivity and higher terrestrial input, while ammonium levels are lower as the water column oxygenates. In summer, total phosphate and nitrate-nitrite levels decrease to fuel increased primary production, which is also fueled by the internal release of phosphorus from sediments during summer anoxia. While a high N:P ratio (> 16:1) can suggest phosphorus limitation, the extreme inputs and abundances in both phosphorus and nitrogen species throughout the year imply that nutrient supply does not control primary production. Other factors such as daylight duration, surface dissolved oxygen saturation, depth of light penetration, and diurnal mixing may play the dominant roles limiting primary productivity.

The SS’s century-long use as a repository for agricultural wastewater has led to predictable but severe ecological consequences. Data compilation presented here reveals a notable decline in dissolved oxygen levels in the water column over the past two decades—a clear sign of advancing eutrophication. While current management efforts prioritize addressing declining inflows and exposed lakebed, our analysis shows that eutrophication fundamentally drives ecosystem deterioration and must be addressed for successful restoration^89^. Even if proposed in-basin strategies such as a flow-through system resolve salinity issues, persistent nutrient enrichment could continue to degrade water quality, making the ecosystem inhospitable for aquatic life and trigger impacts for public health. Moreover, these conditions pose broader environmental and public health risks through the aerosolization of toxins and contaminants in lake spray. Without implementing nutrient reduction strategies alongside water volume and salinity management, restoration efforts cannot achieve meaningful ecological recovery or protect public health in surrounding communities.

The SS presents unique challenges compared to other drying lakes in the arid West, such as the Great Salt Lake, Mono Lake, and Owens Lake. While these systems share water supply challenges, the SS’s extreme nutrient inputs create additional complexities. Our data highlight how nutrient compositions and contamination sources influence biogeochemical dynamics, seasonal cycles, overall water quality, and ecological health. These fundamental water quality issues must be addressed before investing in expensive solutions like desalination technologies^90^ or water importation schemes. We therefore recommend an aggressive top-down approach to reduce external nutrient inputs through policy adjustments and restoration initiatives. Without addressing excessive nutrient loading and its cascading effects on basin redox, productivity blooms, metal cycling, and microbial processes, even successful salinity control measures will fail to restore ecosystem health.

## Methods

### Data sources and collection methods

Lake elevation and salinity data (Fig. 1) were obtained from the USGS (https://www.usgs.gov/special-topics/salton-sea)^91,92^ and US Bureau of Reclamation (USBR) (https://www.usbr.gov/lc/region/programs/saltonsea.html)^35^, respectively. Nutrient Data Surface phosphate and nitrate concentrations for tributaries, tributary mouths (Alamo and New River), and the Salton Sea (Fig. 2 and 4) were compiled from multiple sources: Salton Sea Environmental Timeseries (https://saltonseascience.org/)^32^, Imperial Irrigation District^36^, California Environmental Data Exchange Network (CEDEN)^37^, USBR^35^, Sandia National Lab, field research dataset provided by the Stenstrom lab. While Fig. 2 incorporates data from all sources listed above into a spatial map produced in ArcGIS, Fig. 4exclusively uses only USBR data for the time-monitored seasonal trends. Surface and bottom nutrients (total phosphate, ammonium, nitrate) concentrations were collected by USBR^35^at two sites (SS1: 33.4, −115.925; SS3: 32.266667, −115.758333). Discharge and Flux Calculations Daily discharge data for Alamo and New Rivers were obtained from USGS Westmorland (Site ID 10,255,550)^33^and Niland (Site ID 10,254,730)^34^ gauges, respectively. Nutrient fluxes (Fig. 3) were calculated by multiplying nutrient concentration by monthly average discharge rate. Nutrient analyses conducted by USBR followed EPA certified standard methods: Method 365.1 for ortho-phosphate and total phosphorus, Method 353.2 for nitrate and nitrites, Method 350.1 for ammonium, and Method 351.2 for total nitrogen^93^. Dissolved oxygen measurements (Fig. 5) were collected at the deepest portion of the southern basin (33.26265, −115.739). Pre-2020 data were obtained from USBR^35^. Post-2020 measurements were conducted in situ through the water column using a calibrated YSI EXO2 multi-parameter sonde probe (YSI Incorporated, Yellow Springs, OH, USA). This represents the only temporal trend analysis in our study that bridges two distinct datasets, as other time-series analyses showing seasonal and/or annual trends rely on single-sourced data.

### Data quality assessment

Multiple data sources were evaluated for analytical compatibility and measurement uncertainty. While standardized methods were employed, several potential sources of methodological variation could occur in instrument calibration protocols, laboratory-specific analytical procedures, spatial variations in sampling locations and depths, sample preservation and handling methods, temporal discontinuities between datasets, and differences in method detection limits and reporting conventions. These variations were particularly considered when interpreting dissolved oxygen temporal trends (Fig. 5), which uniquely relied on both USBR^35^ and Lyons lab datasets. While other figures compile data from multiple sources, they either represent spatial patterns rather than temporal trends (Fig. 2) or rely on single-source time series (Figs. 1, 3, 4). Detailed sampling, preparation, and analytical mechanisms beyond EPA methods were not consistently specified across databases, making data interpretation dependent on the accuracy and reproducibility of the compiled public databases.

### Data statistics

Nutrient concentrations and ratios were analyzed using Microsoft Excel (Microsoft 365). To evaluate whether phosphate concentrations exceeded eutrophic thresholds (> 0.05 mg/L), one-tailed Student’s T-tests were performed using the T.TEST function. Probability density functions for N:P ratios were calculated using the EXPON.DIST function to determine the probability of ratios exceeding the Redfield ratio (16:1) in both summer and winter seasons. Dissolved oxygen data were binned into temporal groups (2004–2009, 2010–2019, and 2020–2022) based on water policy milestones rather than equal sample sizes to maintain data integrity while acknowledging temporal gaps. Mean, standard deviations, and sample sizes for each bin are reported in Table 1 to provide context for variability in measurements. Figures were generated using Microsoft Excel for time series analysis and data visualizations and annotated in Adobe Illustrator.

## Data Availability

The data that support the findings of this study are openly available in figshare at https://figshare.com/s/806604c935ac13824c4b.
