# Author Correction: Nutrient loading as a key cause of short- and long-term anthropogenic ecological degradation of the Salton Sea

**DOI:** 10.1038/s41598-025-02749-7

**Published:** 2025-05-27

**Authors:** Caroline Hung, Charles Diamond, Ryan Sinclair, Meng-Chen Lee, Michael Stenstrom, Mara A. Freilich, Quinn Montgomery, Consuelo Marquez, Timothy W. Lyons

**Affiliations:** 1 Department of Earth and Planetary Sciences, University of California, Riverside, CA 92521 USA; 2 School of Public Health, Loma Linda University, Loma Linda, CA 92350 USA; 3 Department of Civil and Environmental Engineering, University of California, Los Angeles, CA 90095 USA; 4 Division of Applied Mathematics and Department of Earth, Environmental, and Planetary Sciences, Brown University, Providence, RI 02912 USA; 5 CA, USA

Correction to: *Scientific Reports* 10.1038/s41598-024-82633-y, published online 28 December 2024

The original version of this Article contained a repeated error, where a unit was incorrect.

As a result, in the Discussion section, under the subheading ‘Other limiting factors for primary production’,

“Phosphate concentrations consistently exceed eutrophic thresholds (> 0.05 mg/L or 0.53 mmol/L), even during summer (Fig. 4).”

now reads:

“Phosphate concentrations consistently exceed eutrophic thresholds (> 0.05 mg/L or 0.53 µmol/L), even during summer (Fig. 4).”

In addition, the units in Fig. 4 were incorrect.

The original Fig. 4 and accompanying legend appears below.

**Fig. 4:**
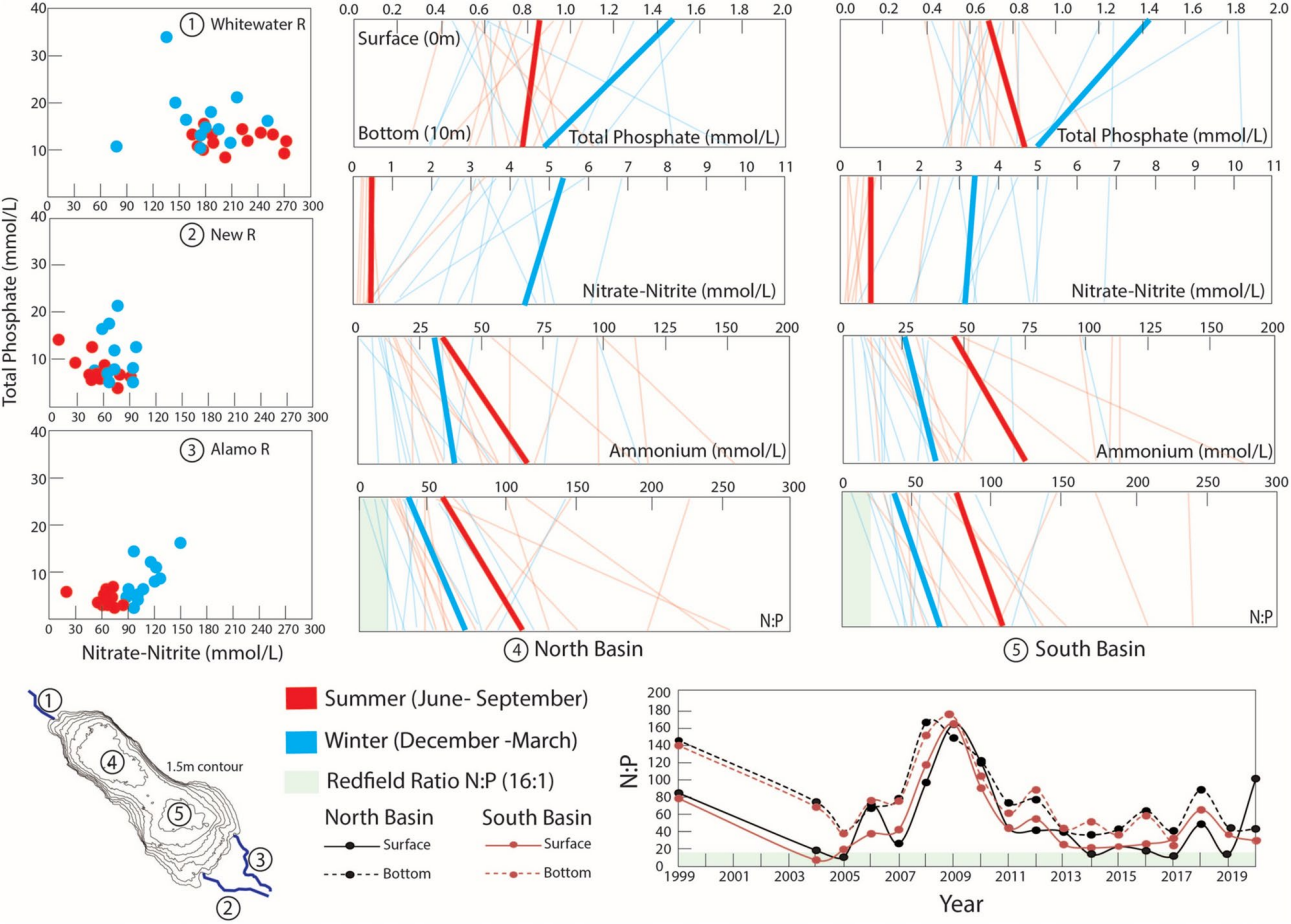
Seasonality of nutrients cycling are shown through: panels 1–3 for nitrate-nitrite (x-axis) and total phosphate (y-axis) concentrations of the Whitewater, New, and Alamo Rivers gathered from independent sampling events from 2004 to 2017; and panels 4–5 for total phosphate, nitrate-nitrite (NO_3_, NO_2_), ammonium (NH_3_) concentrations and the N:P (x-axis) in surface (0 m) and deep (10 m) (y-axis) of the water column in the deepest locale in the north and south basin from 2004 to 2017. The bottom panel shows N:P trends throughout time from 1999 to 2019 in the same locations in the north and south basin. Data from the winter (categorized as sampling dates falling in between 12/21–3/21) are depicted as blue and those from the summer (6/21–9/21) are depicted as red. In panels 4–5, actual concentrations from independent sampling events are shown in lightened lines whereas averages taken from the actual concentrations are shown in bold lines for respective winter and summer periods. Data for the Salton Sea tributaries and water column were gathered from sampling events spanning 2004 to 2017 and are publicly available from the Bureau of Reclamation^35^. Tributary samples were collected at the Whitewater River at 33.52482, − 116.07894 (Lincoln St. intersection). The New River was accessed at 33.08548, − 115.61451 (Gentry Rd. intersection), while Alamo River was accessed at 33.19924, − 115.59710 (along Red Hill Rd. near the Red Hill Marina). The northern and southern deepest locale of the lake was accessed at 33.40013, − 115.92574 and 33.26265, − 115.739, respectively.

The original Article has been corrected.

